# *De novo* transcriptome analysis and identification of genes associated with immunity, detoxification and energy metabolism from the fat body of the tephritid gall fly, *Procecidochares utilis*

**DOI:** 10.1371/journal.pone.0226039

**Published:** 2019-12-17

**Authors:** Lifang Li, Xi Gao, Mingxian Lan, Yuan Yuan, Zijun Guo, Ping Tang, Mengyue Li, Xianbin Liao, Jiaying Zhu, Zhengyue Li, Min Ye, Guoxing Wu

**Affiliations:** 1 State Key Laboratory for Conservation and Utilization of Bio-Resources in Yunnan, Yunnan Agricultural University, Kunming, China; 2 Key Laboratory of Forest Disaster Warning and Control of Yunnan Province, Southwest Forestry University, Kunming, China; Western Sydney University, AUSTRALIA

## Abstract

The fat body, a multifunctional organ analogous to the liver and fat tissue of vertebrates, plays an important role in insect life cycles. The fat body is involved in protein storage, energy metabolism, elimination of xenobiotics, and production of immunity regulator-like proteins. However, the molecular mechanism of the fat body’s physiological functions in the tephritid stem gall-forming fly, *Procecidochares utilis*, are still unknown. In this study, we performed transcriptome analysis of the fat body of *P*. *utilis* using Illumina sequencing technology. In total, 3.71 G of clean reads were obtained and assembled into 30,559 unigenes, with an average length of 539 bp. Among those unigenes, 21,439 (70.16%) were annotated based on sequence similarity to proteins in NCBI’s non-redundant protein sequence database (Nr). Sequences were also compared to NCBI’s non-redundant nucleotide sequence database (Nt), a manually curated and reviewed protein sequence database (SwissProt), and KEGG and gene ontology annotations were applied to better understand the functions of these unigenes. A comparative analysis was performed to identify unigenes related to detoxification, immunity and energy metabolism. Many unigenes involved in detoxification were identified, including 50 unigenes of putative cytochrome P450s (P450s), 18 of glutathione S-transferases (GSTs), 35 of carboxylesterases (CarEs) and 26 of ATP-binding cassette (ABC) transporters. Many unigenes related to immunity were identified, including 17 putative serpin genes, five peptidoglycan recognition proteins (PGRPs) and four lysozyme genes. In addition, unigenes potentially involved in energy metabolism, including 18 lipase genes, five fatty acid synthase (FAS) genes and six elongases of very long chain fatty acid (ELOVL) genes, were identified. This transcriptome improves our genetic understanding of *P*. *utilis* and the identification of a numerous transcripts in the fat body of *P*. *utilis* offer a series of valuable molecular resources for future studies on the functions of these genes.

## Introduction

Crofton weed, *Eupatorium adenophorum* Spreng, is a perennial, hazardous invading species of the family Asteraceae, which is native to Mexico. It has successfully invaded many regions on the globe in a wide variety of natural and anthropogenic ecosystems that range from forest and grassland to farmland [1] and is now one of the most dangerous exotic invasive plants, causing great economic losses and environmental problems worldwide [2]. The tephritid stem gall-forming fly, *Procecidochares utilis* Stone (Diptera: Trypetidae), is an important natural enemy of the noxious invasive *E*. *adenophorum*. The larvae of flies can bore into the *E*. *adenophorum* leading to gall formation, which can inhibit the growth and development of its host plant and can effectively restrict the dispersal of *E*. *adenophorum* [3, 4]. In 1945, *P*. *utilis* was introduced from Mexico to Hawaii to combat *E*. *adenophorum* and it proved an effective biological control agent for *E*. *adenophorum* [5, 6]. Many countries such as New Zealand, Australia, and China have used this insect to control the damage caused by *E*. *adenophorum* [7, 8]. Since *P*. *utilis* is utilized as a biocontrol agent, many of its ecological and biological characteristics have been studied [9], but the molecular research of *P*. *utilis* is relatively limited, and the entire genome is not yet available. The transcriptome sequencing of its alimentary tract of *P*. *utilis* has been performed successfully, and has proved to be an effective method to gather genetic information of the alimentary tract [7]. These data have provided comprehensive gene expression information regarding detoxification. However, the fat body transcriptome has not been investigated.

The insect fat body is a multifunctional organ that fills the adipocytes, which play important roles in the life of insects [10]. The fat body controls the synthesis and use of energy reserves, lipid and glycogen, and has multiple biochemical functions in intermediate metabolism, including lipid, carbohydrate, amino acid and nitrogen metabolism, and protein synthesis [11]. It is a major storage depot for nutrients and fat, and these reserves can be utilized to release energy to meet the energy demands of the insect. Furthermore, the insect fat body participates in the process of immune regulation and production of antimicrobial peptides, which provides an immune barrier between the internal and external environment [12, 13]. In recent studies, a number of genes associated with immune response were identified in transcriptome of the insect fat body. For example, 71, 60 and 44 unigenes encoding various putative immune-related enzymes were identified in *Bactrocera dorsalis*, *Glossina morsitans morsitans* and *Aedes aegypti*, respectively [13–15]. In addition, the insect fat body is also involved in metabolism and detoxification of xenobiotics, harboring several detoxification enzymes [13, 16, 17]. In the *B*. *dorsalis* fat body transcriptome, 37 P450s, 18 GSTs and 29 ABC transporters were identified [13]. With the development of novel next generation high-throughput sequencing technology, the fact that insect fat body serves as a multifunctional organ of great biosynthetic and metabolic importance has also been confirmed by the fat body transcriptomes of *Drosophila melanogaster* [18], the tsetse fly (*G*. *morsitans morsitans*) [14], the yellow fever mosquito (*A*. *aegypti*) [15,19], the Oriental Fruit Fly (*B*. *dorsalis*) [13], the brown planthopper (*Nilaparvata lugens*) [11], the wing polymorphic cricket (*Gryllus firmus*) [20]. Despite the importance of this organ, little is known about its molecular machinery as it relates to its physiology, immunity and energy metabolism. In this study, we performed high-throughput Illumina HiSeqTM ^2000^ to acquire a transcriptome of the fat body for *P*. *utilis*. We mainly focused on genes associated with immune defense, detoxification and energy metabolism, as these genes are important for insect development. The results obtained from this study will improve our genetic understanding of *P*. *utilis*. At the same time, a large number of genes identified in the fat body of *P*. *utilis* will provide a series of valuable resources for further study of the physiological functions of these genes.

## Materials and methods

### Ethics statement

No specific permits were required for the insects collected in this study. The *P*. *utilis* used for this study is neither an endangered or protected species. Galls of *E*. *adenophorum* were collected in Kunming, China and no specific site permission was required for this location as it is not privately-owned or protected.

### Insect samples

Galls of *E*. *adenophorum* were originally collected from Kunming, Yunnan Province, China. All galls were kept in cages and reared under laboratory conditions at 25°C and 75% relative humidity as described previously [8]. Fresh galls were dissected with a scalpel and then larvae were collected for later experiments.

### Fat body collection and RNA isolation

Fat body samples of *P*. *utilis* were obtained from third instar larvae. Firstly, 75% alcohol was used to disinfect the skin of *P*. *utilis* larvae, then skins of the larvae were dissected from head to tail and the Malpighian tubules, cuticula, salivary glands, midgut and other residues were discarded. The fat body samples were transferred to an Eppendorf tube with Trizol on ice. They were stored at -80°C until RNA extraction.

Total RNA was isolated from the fat bodies using the Trizol reagent (Invitrogen, USA) according to the manufacturer’s instructions. The concentration and quality of total RNA were determined by an Agilent 2100 Bioanalyzer (Agilent RNA 6000 Nano Kit, Agilent Technologies, USA) and NanoDrop^TM^ spectrophotometer (Thermo Fisher, Waltham, MA, USA), respectively.

### cDNA library preparation and Illumina sequencing

The cDNA libraryof *P*. *utilis* fat body was constructed from a pool of 100 fat bodies using the mRNA-Seq Sample Preparation Kit (Illumina, San Diego, CA USA) following the manufacturer’s instruction. In brief, the Poly (A) mRNA was purified from 20 μg of total RNA using Oligo (dT) magnetic beads. It was then chemically fragmented into short sequences in the presence of fragmentation buffer at 94°C for 5 min. These short sequences were used as templates to synthesize first-strand cDNA using random hexamer-primers. Subsequently, second-strand cDNAs were synthesized using buffer, dNTPs, RNaseH and DNA polymerase IDNA polymerase I (New England BioLabs, Ipswich, MA). After that, these cDNA short fragments were purified using QiaQuick PCR extraction kit (Qiagen) and then resolved with EB buffer for end reparation and single nucleotide A (adenine) addition. Finally, the cDNAs were connected with sequencing adapters. Suitable fragments (200–250 bp), as judged by agarose gel electrophoresis, were collected and used as templates for PCR amplification. The quantification of cDNA library was validated and quantified via Agilent 2100 bioanalyzer and ABI StepOnePlus Real-Time PCR System. The mean read lengths of the cDNA library were 350 bp. Finally, the cDNA libraries (three biological replicates) were pooled and sequenced in one lane on the Illumina HiSeq^TM^ 2000 platform using paired-end technology in a single run at Beijing Genomics Institute (BGI; Shenzhen, China). A total of 45,639,322 raw reads were obtained and the raw transcriptome data was deposited in the NCBI Short Read Archive (SRA) with the accession number: SRR8521423.

### Transcriptome *De novo* assembly and bioinformatics analysis

Prior to assembly, reads with adaptors, the number of reads with unknown nucleotides content larger than 5% and low quality reads which the percentage of low quality bases (base quality≤10) was more than 20% were removed from raw data by using filter_fq (BGI internal software). Meanwhile the clean data were calculated based on Q20, Q30, GC-content and sequence duplication level. The subsequent sequencing on Illumina HiSeq^TM^ 2000 for paired read of 100 or 105 bases following manufacturer’s manual. The clean reads were then *de novo* assembled into unigenes using Trinity v2.0.6 with a Kmer_length of 25 and all other parameters set to default [21]. Next, TIGR Gene Indices clustering tools (TGICL) was used to perform sequence clustering and generate unigenes [22]. Briefly, clean reads with a certain length of overlap (the overlap length between k-mer was equal to k-1 to extend the seed until it could no longer be extended) were combined to form contigs. Then, using paired-end reads, reads were then mapped back to the contigs and contigs from the same transcript, as well as the distances between these contigs were detected. Trinity connected these contigs to produce sequences that could not be extended on either end, were considered unigenes. After clustering, the obtained unigenes were divided into two classes: clusters, with the prefix CL, and singletons, having the prefix unigene.

After assembly, homology searches and annotation of all unigenes were performed using the BLASTn programs against nucleotide sequence databases (Nt, e-value≤ 1.0e^−5^) in NCBI [23]. Unigenes were also aligned by BLASTx with the non-redundant protein (Nr, e-value≤ 1.0e^−5^), euKaryotic Ortholog Groups (KOG), Kyoto Encyclopedia of Genes and Genomes (KEGG) and Swiss-Prot databases [23]. Functional annotation by Gene Ontology (GO) terms (http://www.geneontology.org) was analyzed by Blast2GO v2.5.0 software with e-value less than 1.0e^−6^ [24] and InterPro was performed by InterProScan5 [25].

### Identification and analysis of interesting genes

Sequences putatively encoding genes related to detoxification, immunity and energy metabolism, such as detoxification enzymes (GSTs, CarEs, P450s and ABC transporters), immune-related enzymes (serpin, PGRP and lysozyme) and energy metabolism enzymes (lipase, FAS and ELOVL) were identified by the BALSTx alignment against the nr database with a cut-off E-value of 10^−5^. Contigs from the same cluster represent the same unigene. If a cluster contains several contigs, the longest one was selected for further analysis. The full open reading frames of interesting genes were determined (http://www.ncbi.nlm.nih.gov/gorf/gorf.html) and were further verified by protein BLAST results. Amino acid sequences were aligned using the Clustal W. The interesting gene families in *P*. *utilis* were compared to different model insect species, *D*. *melanogaster*, *B*. *dorsalis* and *C*. *capitate*. The GST, CarE, P450, ABC transporter, serpin, PGRP, lysozyme, lipase, FAS and ELOVL sequences of *D*. *melanogaster*, *B*. *dorsalis* and *C*. *capitate* were downloaded from NCBI. Subsequently, the phylogenetic trees were constructed based on the amino acid sequence alignment using Neighbor-joining (NJ) method in software MEGA 5 [26]. Bootstrap analysis of 1,000 replication trees was performed to evaluate the branch strength of each tree.

## Results and discussion

### Illumina sequencing and de novo assembly

**Fig 1:**
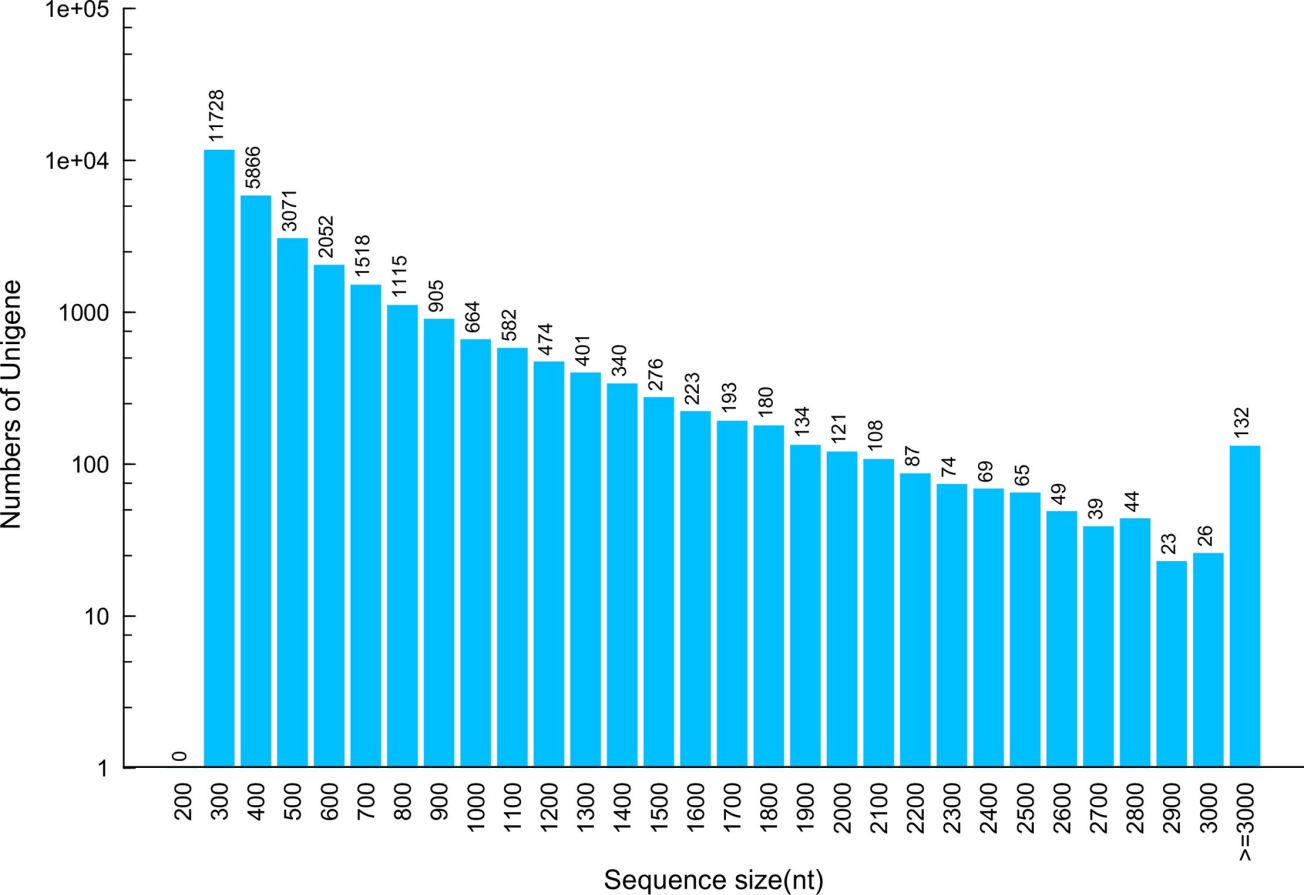
Length distribution of unigenes in transcriptome assembly for *P*. *utilis* fat body.

Illumina sequencing was used to sequence a cDNA library of the *P*. *utilis* fat body, and generated about 45.64 Mb raw reads. After filtration, a total number of 41,241,514 clean reads were assembled into 40,467 contigs with a total length of 19,806,004 bp and a mean length of 489 bp. The contigs were further assembled into 30,559 unigenes with an average length of 539 bp (Table 1), which is shorter than that obtained from the *N*. *lugens* (avirulent TN1 population with a mean length of 656 bp and the virulent Mudgo (M) population with a mean length of 676 bp) [11] and *B*. *dorsalis* [13]. Among all unigenes, 4,304 unigenes (14.08%) were longer than 1,000 bp, 9,325 unigenes (30.5%) were longer between 500 and 1,000 bp, and 20,665 unigenes (67.6%) ranged from 300 to 500 bp (Fig 1).

**Table 1:** Sequencing summary for the fat body transcriptome of *P*. *utilis*.

Sequencing Summary	Fat body specific transcriptome
Total raw reads(Mb)	45.64
Total clean reads	41,241,514
Total clean bases (Gb)	3.71
Q20 percentage (%)	98.72
Q30 percentage (%)	94.19
Number of contigs	40,467
Total length of contigs (bp)	19,806,004
Mean length of contigs (bp)	489
N50 length of contigs (bp)	623
N70 length of contigs (bp)	362
N90 length of contigs (bp)	232
GC percentage (%)	39.53
Number of unigenes	30,559
Total length of unigenes (bp)	16,485,836
Mean length of unigenes (bp)	539
N50 length of unigenes (bp)	685
N70 length of unigenes (bp)	403
N90 length of unigenes (bp)	255
GC percentage (%)	39.84
Total number of complete ORFs	2,808
Total number of partial ORFs	11,164

### Functional annotation of the *P*. *utilis* fat body unigenes

**Fig 2:**
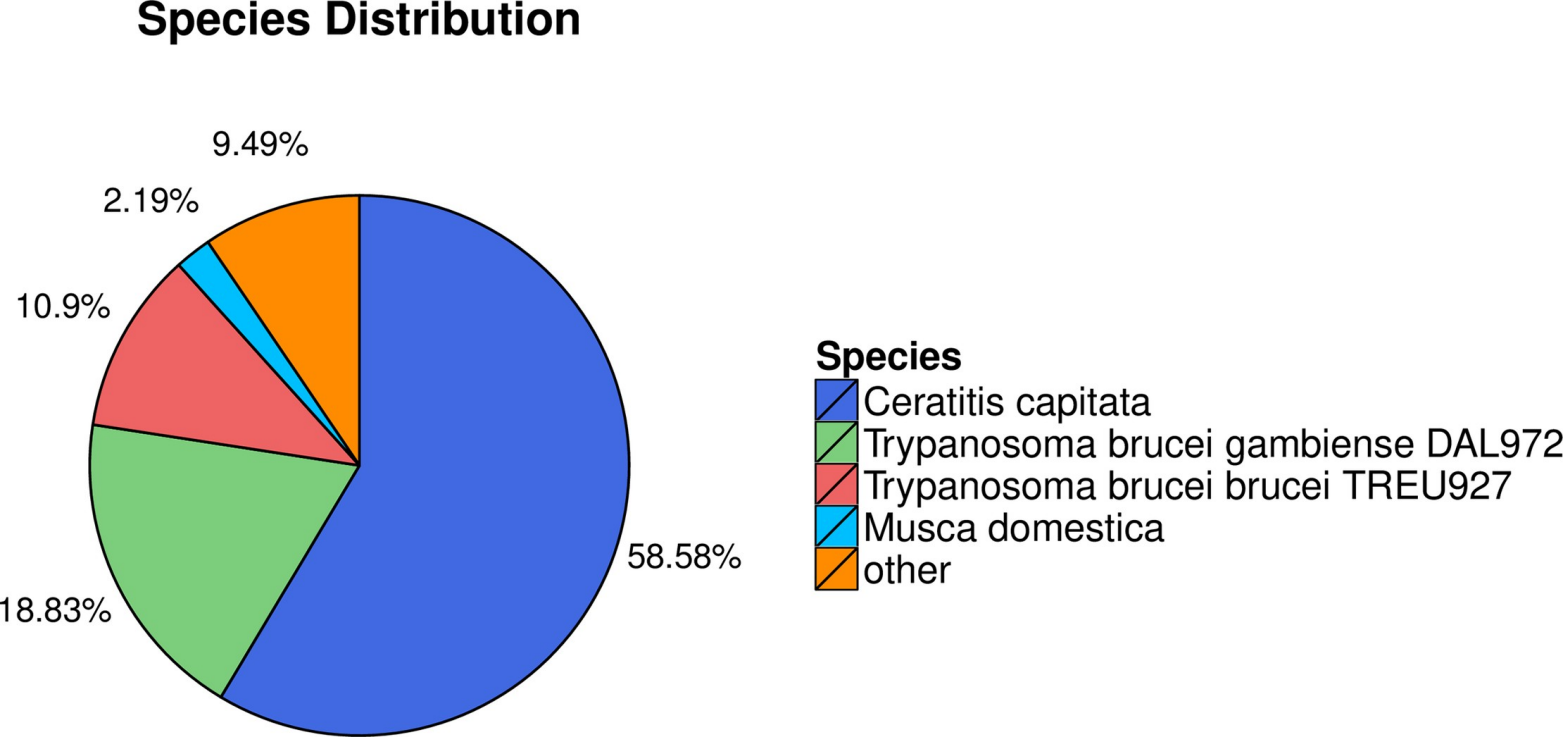
Species distribution of the top BLASTX matches to the *P*. *utilis* fat body unigenes.

For functional annotation, a total of 30,559 unigenes were searched against the Nr, Nt, SwissProt, KEGG, KOG, InterPro and GO databases. The results showed that 21,439 (70.16%), 13,657 (44.69%), 12,188 (39.88%), 12,405 (40.59%), 12,925 (42.30%), 13,149 (43.03%) and 9,354 (30.61%) unigenes matched to Nr, Nt, SwissProt, KEGG, KOG, InterPro and GO known protein databases, respectively (Table 2). After Nr database annotation, the species distributions demonstrated that 58.58% of the unigenes had best matches against sequences of *Ceratitis capitata*, followed by *Trypanosoma brucei gambiense* DAL972 (18.83%), *Trypanosoma brucei brucei* TREU927 (10.9%) and *Musca domestica* (2.19%) (Fig 2). Even though a large percentage of transcripts matched to trypanosomes, PCR analysis confirmed the lack of contamination in *P*. *utilis* fat bodies and thus, the transcripts are likely not contaminants and are likely truly derived from the fat bodies of *P*. *utilis* (S1 Text). The species distribution was consistent with our previous study of transcriptome analysis of the alimentary tract of *P*. *utilis*, in which more than 50% of genes were most matched to *C*. *capitate* [27]. However, in the fat body of *B*. *dorsalis*, the highest percentage of unigene sequences were matched with *Drosophila* (over 79%) [13].

**Table 2:** Number of unigenes annotated in seven databases.

Database	The number of unigenes	Percentage (%)
Nr	21,439	70.16
Nt	13,657	44.69
Swissprot	12,188	39.88
KEGG	12,405	40.59
KOG	12,925	42.30
Interpro	13,149	43.03
GO	9,354	30.61
Total number of unigenes	30,559	100
All databases	2,692	8.81
At least one database	24,395	79.83

**Fig 3:**
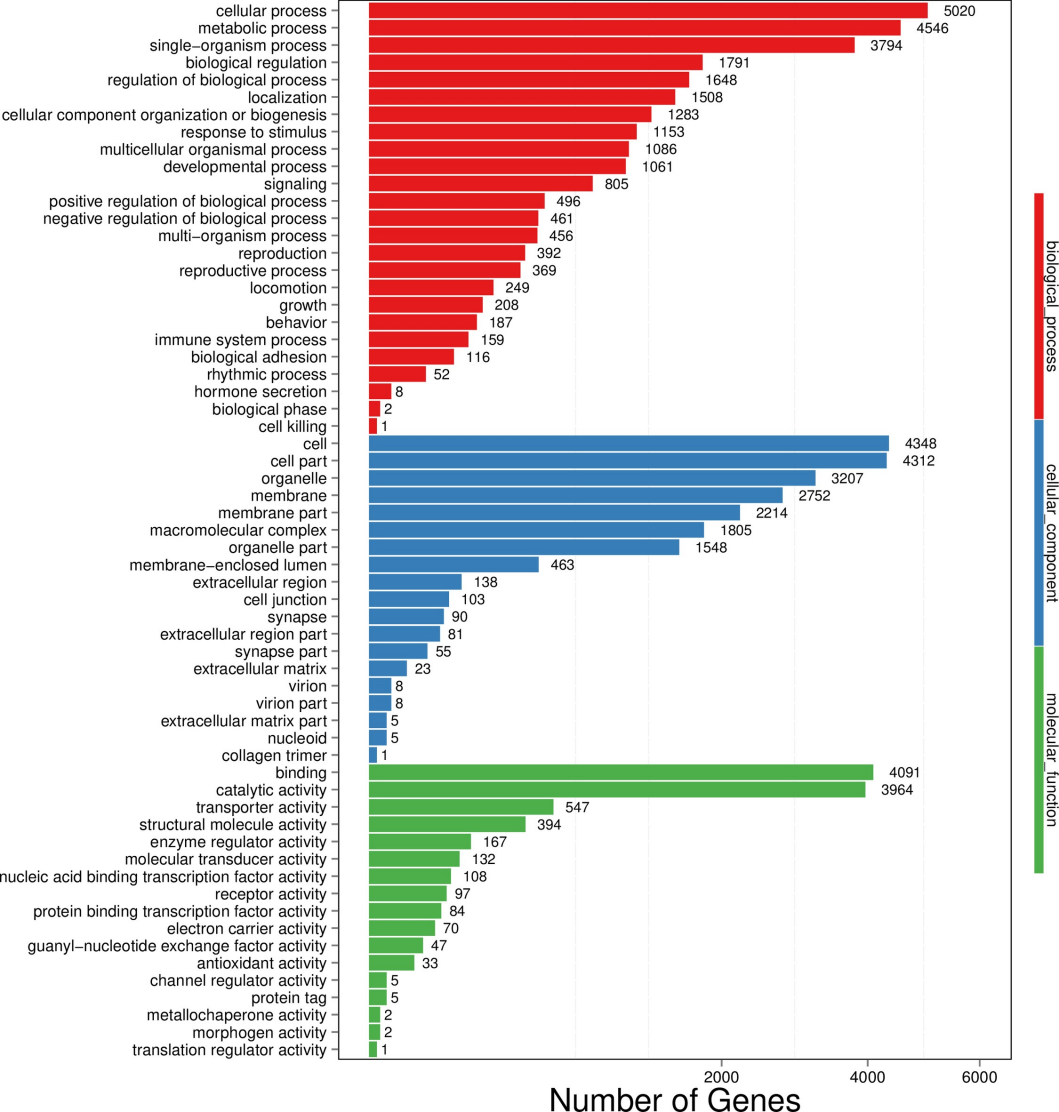
Classification of the gene ontology (GO) for the transcriptome of the fat body from *P*. *utilis*.

Gene ontology (GO) assignments were used to functionally classify the predicted unigenes from the fat body of *P*. *utilis*. According to the sequence similarity, 9354 unigenes (30.61%) were annotated and classified into 61 functional groups of three main ontologies: biological processes (46.48%) was the largest category, followed by cellular components (16.88%) and molecular function (36.64%) (Fig 3). In the biological processes category, “cellular process” and “metabolic process” terms were the two largest groups. In the cellular components category, “cell” and “cell parts” terms were the most abundant. In the molecular function, “binding” and “catalytic activity” terms were the two largest groups. The results were similar to the transcriptome study of the fat body from *N*. *lugens* [13]. Meanwhile, the “cell killing” term in the biological processes category was the smallest group, containing only one unigene. Only one unigene each was assigned in the functional groups “cell killing”, “collagen trimer” and “translation regulator activity” (Fig 3).

**Fig 4:**
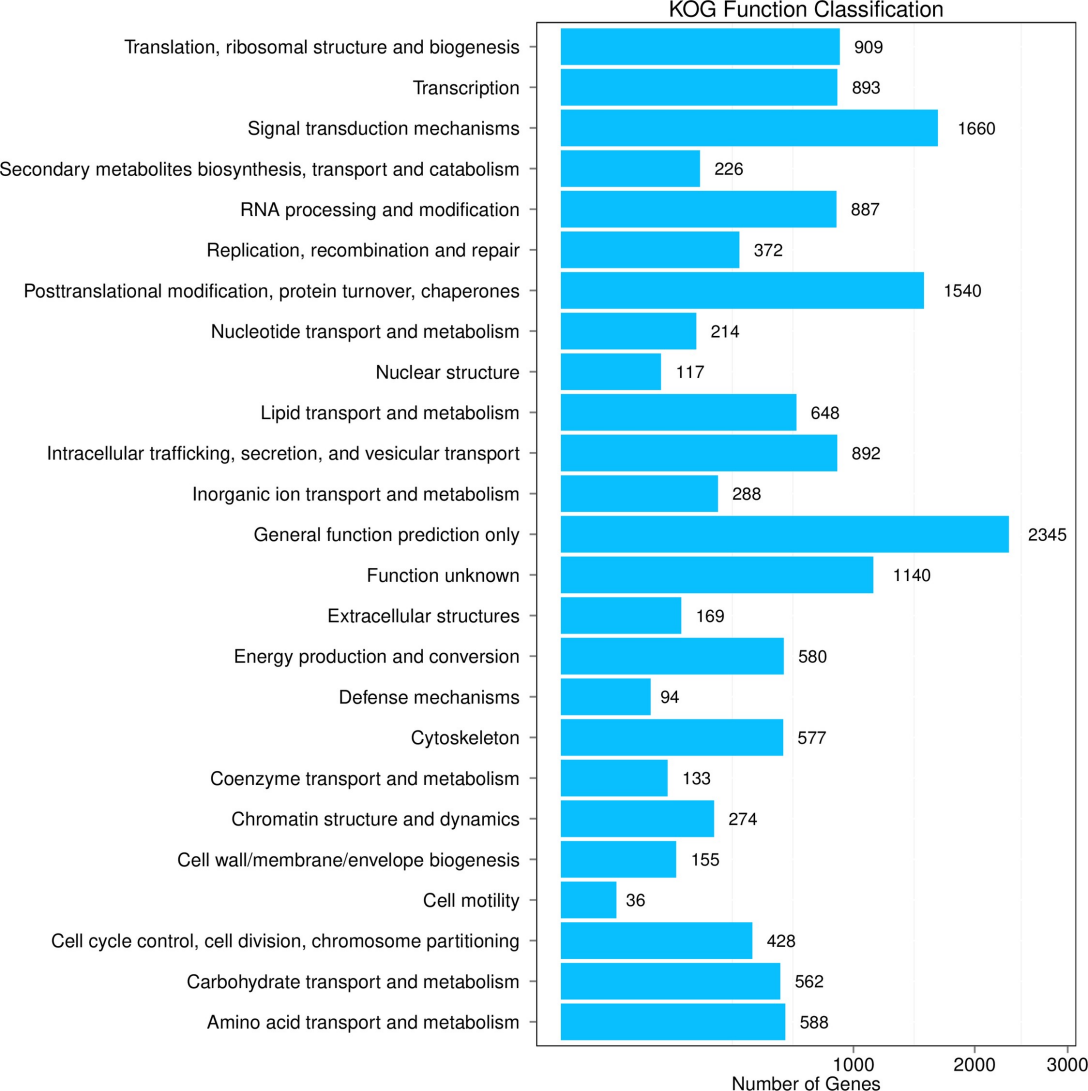
KOG classification of the fat body transcriptome of *P*. *utilis*.

In addition, to obtain more detailed annotation information of the transcriptome, all unigenes were compared against the KOG for functional prediction and classification. In all, 12,925 (42.30%) unigenes were annotated and classified into 25 KOG categories (Fig 4). Among all these categories, the “General function prediction only” contained 2,345 unigenes, which was the largest category, followed by “Signal transduction mechanisms” (1660), “Posttranslational modification, protein turnover, chaperones” (1,540) (Fig 4). Among these unigenes, 1,140 were classified as“function unknown” categories, which revealed that these may be unique or novel genes in the fat body transcriptome of *P*. *utilis*. In contrast, “Defense mechanisms” and “Cell motility” represented relatively smaller KOG groups, containing 94 and 36 unigenes, respectively (Fig 4).

**Fig 5:**
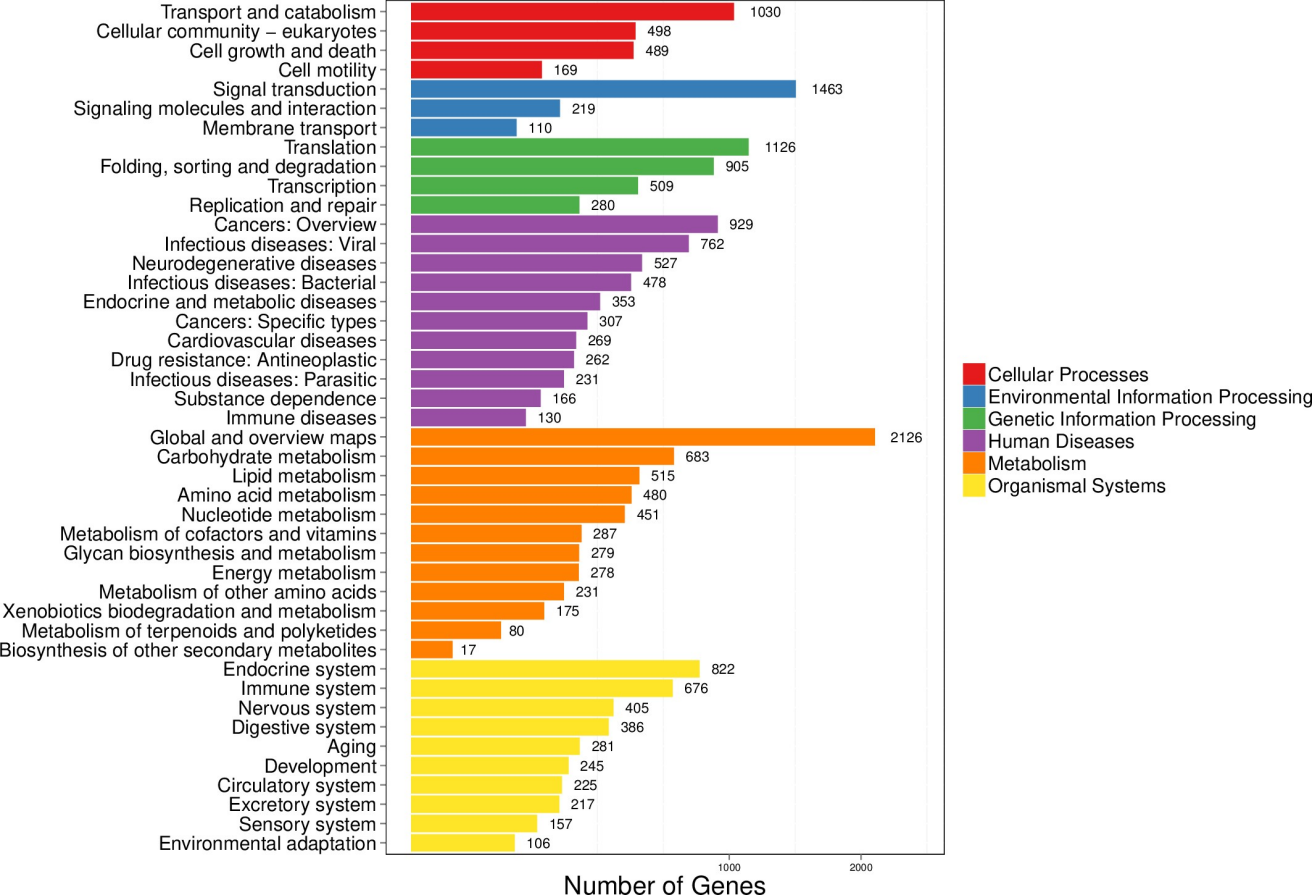
KEGG pathway classification of unigenes in the fat body of *P*. *utilis*.

Subsequently, the KEGG pathway assignment was also performed on unigenes to identify the biological pathways including metabolic and regulatory pathways that will be actively involved in the fat body of *P*. *utilis*. In total, 12,405 unigenes (40.59%) were annotated to the KEGG database and mapped to the cellular processes, environmental information processing, genetic information processing, human diseases, metabolism, and organismal systems pathways (Fig 5). Among these pathways, the most abundant pathway is the global and overview maps (2,126, 17.14%), followed by signal transduction (1,436, 11.58%), translation (1,126, 9.08%), and transport and catabolism (1,030, 8.3%) (Fig 5). The KEGG pathway assignment will be helpful to further research specific biological processes, functions and pathways that exist in the fat body of *P*. *utilis*.

### Simple sequence repeats discovery

**Fig 6:**
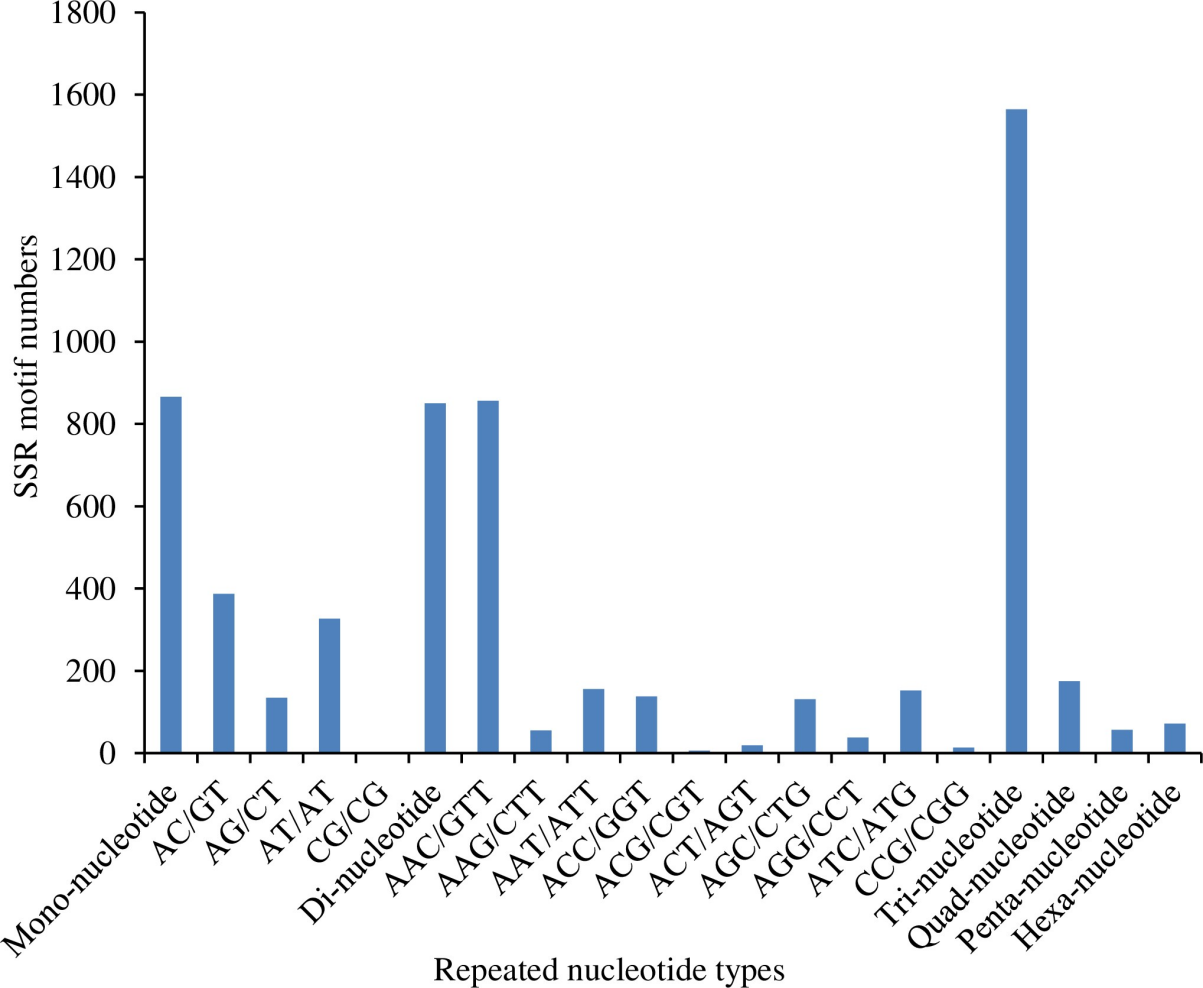
Statistics of SSR nucleotide classes found in the transcriptome of *P*. *utilis* fat body.

Based on the unigene sequences, a total of 2,839 sequences containing 3,585 SSRs were identified from 30,559 unigenes. Among all SSRs, 544 sequences contained more than one SSR, and trinucleotide repeats (43.65%) represented the largest proportion of microsatellite repeat units, followed by mononucleotide (24.16%), dinucleotide (23.7%), quadranucleotide (4.88%), hexanucleotide (2.00%) and pentanucleotide (1.59%) repeats (Fig 6). Among all repeat types, A/T (24.1%), AAC/GTT (23.88%) and AC/GT (10.79%) are the most abundant ones (S1 Table). In total, 3,585 SSRs were identified. The SSRs identified in this research will be helpful for further studies related to population genetic structure in *P*. *utilis*, such as genetic variation and gene flow.

### Identification of the major detoxification-related genes

The insect fat body is an endocrine organ, which involved in metabolism and detoxification of xenobiotics, harboring several detoxification enzymes [13, 16, 17]. In this study, we obtained abundant fat body transcriptome data of *P*. *utilis*, and identified many unigenes that are highly similar to those related to xenobiotic metabolism in other insects, including 50 cytochrome P450 (P450s), 18 glutathione S-transferases (GSTs), 35 carboxylesterases (CarEs), 26 ATP-binding cassette (ABC) transporters.

#### Cytochrome P450s

**Fig 7:**
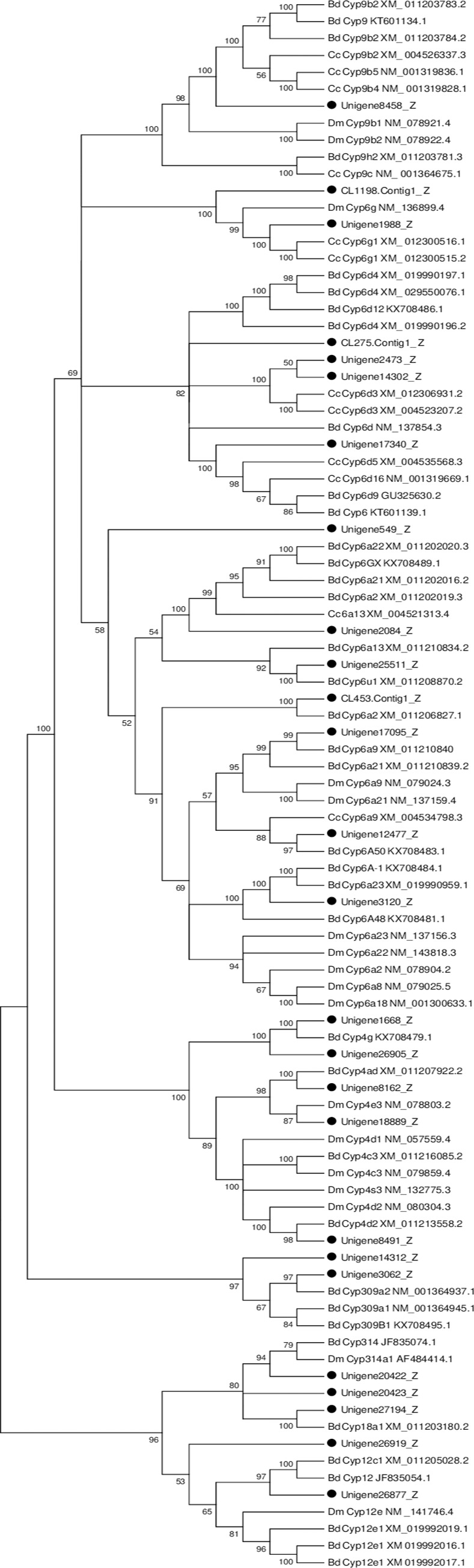
Neighbor-joining phylogenetic tree of *P450* genes from the *P*. *utilis* fat body (●) and other insects. Numbers at each branch node represent bootstrap values.

Cytochrome P450 (P450s) phase I enzymes constitute an old and widely distributed protein superfamily that participated in the metabolism and detoxification of a wide variety of plant secondary metabolites and pesticides [28, 13]. The functional and evolutionary diversity of P450s play key roles in insect adaptation to various ecological environments [29]. They are divided into four clades, CYP2, CYP3, CYP4 and mitochondrial. To date, P450s have been identified from many insects (Table 3) [29–31]. In this study, a total of 50 unigenes corresponding to P450s were identified in the *P*. *utilis* fat body transcriptome based on the Nr annotation results. The number was less than the number of CYP450s identified in the fat body transcriptome of *B*. *dorsalis*. According to the closest blast match in the NCBI nr database, all the identified P450s were divided into four clades: CYP2 (1), CYP3 (30), CYP4 (11) and the mitochondrial CYP clade (5) (S2 Table). After removing short sequences, 26 unigenes were used for phylogenetic analysis. The results showed that these unigenes were divided into seven families: Cyp9 (1), Cyp6 (13), Cyp4 (5), Cyp12 (2), Cyp309 (2), Cyp314 (2) and Cyp18 (1) (Fig 7). The majority of the P450s in *P*. *utilis* fat body belong to the Cyp6 family and Cyp4 family. In our previous studies, 22 unigenes were assigned to eight families according to phylogenetic analysis. Five, five, four, three and two genes belonged to the Cyp 4, Cyp 9, Cyp 6, Cyp 307 and Cyp 12 families, respectively. Lastly, single unigene expression in the alimentary canal were identified as members of Cyp 315, Cyp 314, and Cyp 302 families and most of *P450* unigenes in the alimentary tract of *P*. *utilis* belong to Cyp4, Cyp6 and Cyp9 families. By comparing these unigenes identified in the fat body and alimentary tract of *P*. *utilis*, 17 and 12 unigenes were unique to the fat body and alimentary tract, respectively, and nine unigenes were found in both tissues [27].

**Table 3:** Number and class distribution of P450s genes in other insects.

Classification	Pu	Dm	Ss	Gm	Bm	Tc	Ag	As
CYP2 clan	1	7	6	14	7	8	10	8
CYP3clan	30	36	44	30	28	79	40	44
CYP4 clan	11	32	19	18	33	47	46	34
Mitochondrial clan	5	12	10	15	10	9	9	7
Total	50	87	79	77	78	143	105	93

Pu, *P*. *utilis*; Dm, *D*. *melanogaster*; Ss, *Shirakiacris shirakii*; Gm, *Grapholita Molesta*; Bm, *Bombyx mori*; Tc, *T*. *castaneum*; Ag, *A*. *gambiae*; As, *Anopheles sinensis*. Data of Dm, Gm, Bm, Tc, Ag from Guo *et al* (2017). Data of Ss from Qiu *et al* (2016). Data of As from Zhou *et al* (2015).

In previous studies, most of the P450s belong to the CYP3 and CYP4 clades. These clades in insects are involved in xenobiotic metabolism, such as pesticides and plant secondary metabolites [32–37]. For instance, three of the four CYP6F subfamily genes and *CYP9AQ2* in *Locusta migratoria* were related to the detoxification of deltamethrin or carbaryl [38, 39]. *CcCYP6A51* in *C*. *capitata* was associated with the detoxification of lambda-cyhalothrin [40]. *CYP4g1*, *CYP6g1* and *CYP12d1* confer DDT resistance in *D*. *melanogaster* [41]. In *Plutella xylostella*, *CYP6BG1* conferred resistance to pyrethroid [42], recently, overexpression of *contigs00326* and *02103*, both members of the CYP6 P450 family were known to be associated with pyrethroid detoxification in *Bactrocera oleae* [43]. Moreover, the expression levels of P450s were differentially induced by feeding on the different host plants of herbivorous insects. *Oedaleus asiaticus* grasshoppers feeding on *Artemisia frigida* (a species with low nutrient content and a high level of secondary compounds), had higher P450s enzyme activity and the gene expression level of cytochrome *P450 6K1* was highest compared to feeding on the grasses *Cleistogenes squarrosa*, *Leymus chinensis*, and *Stipa krylovii*, that level showed a significant positive correlation with the secondary compounds of host plant [33]. *Rhynchophorus ferrugineus* larvae fed on diet containing methyl eugenol, methyl isoeugenol, and rosmarinic acid tremendously enhanced the expression of P450 gene [32, 44]. Several CYP6AS subfamily genes and *CYP9Q1* in *Apis mellifera* were related to the detoxification of quercetin, a flavonol widely present in the honey bee’s distinctive diet of honey and bee bread [45, 46]. Moreover, in *A*. *mellifera CYP9Q2*, and *CYP9Q3* detoxify both tau-fluvalinate and coumaphos; these twogenes can also be up-regulated by quercetin [47]. Furthermore, the expression of *CYP6AB14*, *CYP321A7* and *CYP321A9* genes of *Spodoptera litura* were induced by the toxic allochemicals xanthotoxin, coumarin, and flavones [48]. Likewise, the expression of *CYP6A8*, *CYP6D5*, *CYP6W1*, *CYP9B2*, and *CYP12D1* of *D*. *melanogaster* were induced by *Piper nigrum* extracts, and *CYP6A2* and *CYP6A8* expression was induced by caffeine [49, 50]. It is possible that during the evolution, *P*. *utilis* has produced P450s to adapt to the toxins of its host plant *E*. *adenophorum*.

#### Glutathione S-transferases

**Fig 8:**
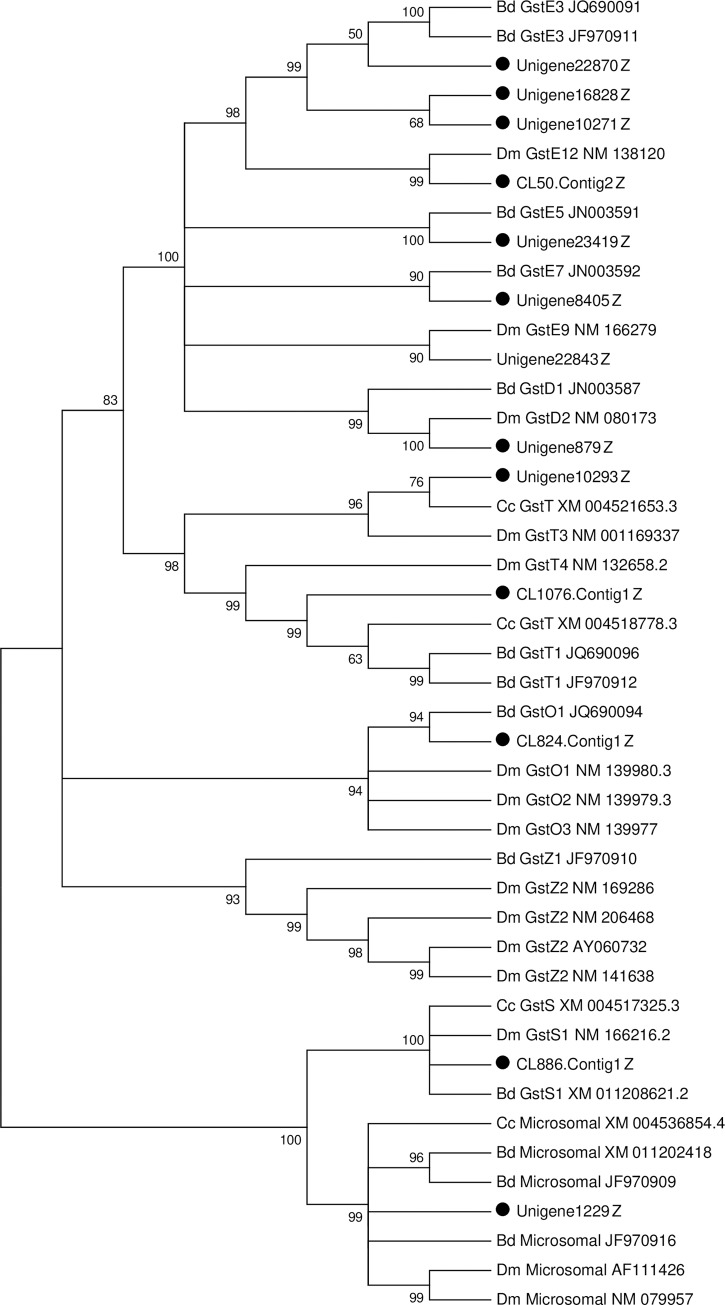
Neighbor-joining phylogenetic tree of glutathione S-transferase (GST) genes from the *P*. *utilis* fat body (●) and other insects. Numbers at each branch node represent bootstrap values.

Glutathione S-transferases (GSTs) are multifunctional enzymes that play a crucial role in phase II detoxification of endogenous and xenobiotic compounds including plant secondary metabolites and pesticides [51]. In insects, GSTs are ordinarily divided into several major subclasses: Delta, Epsilon, Sigma, Omega, Theta, Zeta, Microsomal and others [52, 53]. In particular, Delta and Epsilon were two insect-specific classes and have been widely associated with responses of environmental stress, especially during xenobiotic detoxification [54, 55]. In *D*. *melanogaster*, *B*. *dorsalis*, *G*. *molesta* and *Bactrocera minax* 37, 14, 28, 27 GSTs genes have been identified [30, 56–58]. In this study, a total of 18 sequences encoding GSTs were identified in the fat body of *P*. *utilis* (S3 Table). After removing short sequences, 13 GSTs unigenes were manually selected for phylogenetic analysis. The results showed that these unigenes were assigned to six classes: seven unigenes belonged to the Epsilon class, one unigene belonged to the Delta class, and two unigenes belonged to the Theta class, one unigene was assigned to each of the Omega, Sigma and Microsomal GST classes (Fig 8). No genes belonged to the Zeta class. Most of the genes belonged to Epsilon class, which is in agreement with the previous studies from the fat body of *B*. *dorsalis* [13]. In our previous analysis of the alimentary canal transcriptome, 21 unigenes were used for phylogenetic analysis after removing overly short sequences. The result indicated that they belonged to seven families: Epsilon (8), Delta (4), Theta (2), Zeta (1), Omega (1), Sigma (1), and Microsomal (4) families, and like the fat body, most of the unigenes were assigned to the Epsilon family. Interestingly, by comparing these unigenes identified in the fat body and alimentary canal of *P*. *utilis*, eight unigenes were unique to fat body, 13 unigenes were unique to alimentary canal, and five unigenes were found in both tissuse.

It has been demonstrated that Delta and Epsilon two classes of GSTs were unique in insect species and responsible for detoxification and adaptation to environmental selection pressures [57, 59]. For example, in *B*. *dorsalis*, three Epsilon GSTs have been implicated in resistance to malathion and overexpression of the genes increased the detoxification of malathion in *B*. *dorsalis* [30, 60]. The *LmGSTd1* gene might be related to the resistance of chlorpyrifos in *L*. *migratoria*, and the expression of this gene was induced by chlorpyrifos [61]. The expression level of *Drosophila GSTD2* and *GSTD5* genes were upregulated after exposure to the heavy metals, cadmium and zinc [62]. In addition, some studies suggest that *Metaseiulus occidentalis* and *Tetranychus urticae* Microsomal GSTs may be involved in eliminating toxic xenobiotics, protection against oxidation and pesticide resistance [63–65]. In *M*. *occidentalis* and *B*. *mori*, the Zeta class GSTs not only participated in the oxidative stress response and catalyzed the degradation of tyrosine and phenylalanine, but they also probably played a role in pesticide resistance [63, 66, 67]. The Sigma GSTs of *B*. *dorsalis*, *D*. *melanogaster* and *S*. *litura* play a role in detoxification of toxic compounds and oxidative stress resistance and may also be involved in muscle structure [13, 58, 68]. Moreover, the GSTs have remarkable connections with the secondary compounds in the host plant. The GSTs of *Hyphantria cunea* play important roles in degrading plant secondary metabolites [69]. *Rhynchophorus ferrugineus* larvae fed on diet containing α-asarone, eugenol, coniferyl aldehyde, rosmarinic acid, methyl isoeugenol and methyl eugenol enhanced the expression of GSTs [32, 70]. The *P*. *utilis* GSTs unigenes in these classes may play a similar role as their counterparts in other insect species.

#### Carboxylesterases

Carboxylesterases (CarEs) is a multigene family and ubiquitous in animals, plants, insects, and microbes, which play an important role in insecticide resistance, allelochemical metabolism and tolerance, defense, and regulating development [71]. For example, CarEs are involved in metabolism of phenolic glycosides in *Papilio canadensis* [72]. The CarEs of *Anoplophora glabripennis* play important roles in promoting *A*. *glabripennis* colonization and survival in trees that produce phenolic glycosides in the family Salicaceae [73, 74]. Furthermore, CarEs are also implicated in neurogenesis, hormones and pheromones degradation [75]. Insects CarEs can be divided into 13 clades. These clades fall into three main groups, A-C clades belong to the dietary/detoxification group, D-G clades belong to the hormone/semiochemical processing group, and I-M clades belong to the neuro/developmental group [76]. 35, 24, 44 CarEs have been identified in the genome of *D*. *melanogaster*, *A*. *mellifera* and *Metaseiulus occidentalis* respectively [63, 77], while 15 and 38 putative CarEs have been identified in the transcriptome of *B*. *oleae* and *B*. *dorsalis* [76, 78]. In this study, 35 putative CarEs have been identified in the fat body transcriptome of *P*. *utilis* (S4 Table).

**Fig 9:**
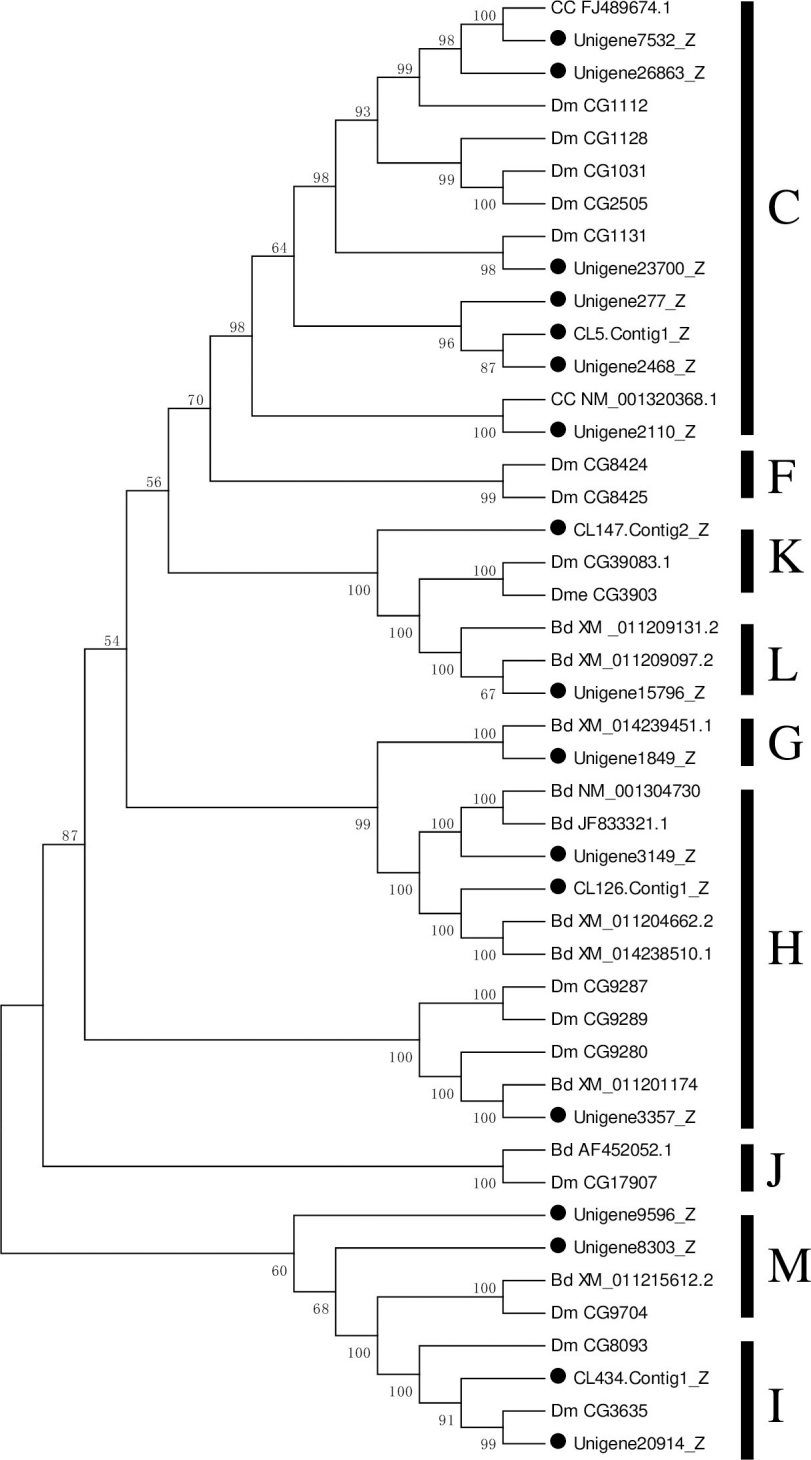
Neighbor-joining phylogenetic analysis of CarE genes from the *P*. *utilis* fat body (●) and other insects. Numbers at each branch node represent bootstrap values.

Based on phylogenetic analysis with other known insect CarEs, 17 CarEs identified in the fat body transcriptome of *P*. *utilis* were divided into seven clades: seven in clade C (α-esterases), three in clade H (glutactins and glutactin-like enzymes), two in each M (neurotactins) and I (uncharacterized) clade, one in each K (gliotactin), L (neuroligins), G (lepidopteran JhE). No CarEs in the fat body transcriptome of *P*. *utilis* belonged to A, B, D, E, F and J clades (Fig 9). Interestingly, 12 of these unigenes coding for CarE were unique to the fat body and were not detected in our previous analysis of the alimentary canal [27]. In this study, most of the CarEs (seven α-esterases) belong to clade C of the dietary detoxification group. This phenomenon was consistent with the CarEs clade in *L*. *migratoria* and *S*. *shirakii* [79, 31]. This finding is consistent with that described in *L*. *migratoria* and *S*. *shirakii*, a large number of detoxification genes can be used to detoxify many different plant secondary metabolites and to develop insecticide resistance [79].

**Fig 10:**
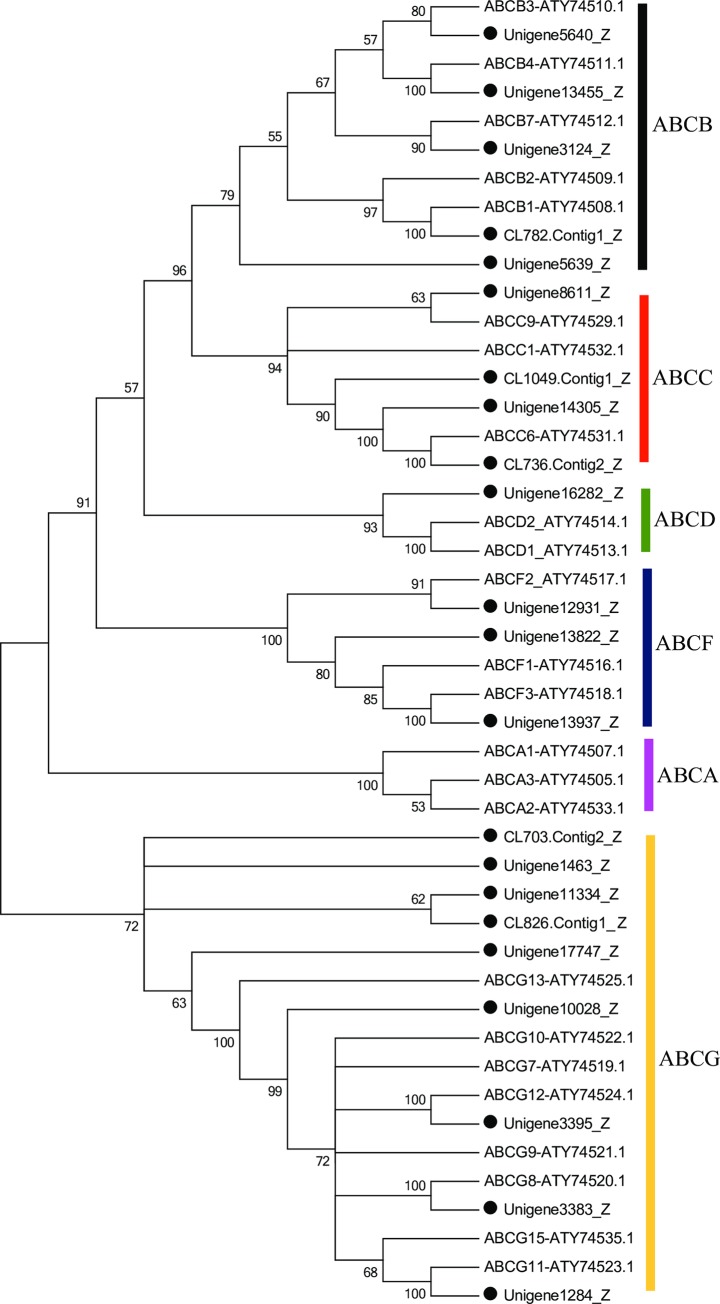
Neighbor-joining phylogenetic analysis of ABC genes from the *P*. *utilis* fat body (●) and *B*. *dorsalis*. Numbers at each branch node represent bootstrap values.

The ATP-binding cassette (ABC) transporters are membrane-bound proteins belonging to the ABC superfamily located in the cellular membrane in all living organisms from bacteria to humans [80, 81]. ABC transporters have been subdivided into eight subfamilies, from ABC-A to ABC-H [82, 83]. In insects, ABC transporters are not only involved in the molecule transport of molecules, but also play a major role in the metabolism of xenobiotics [76]. From the fat body transcriptome of *P*. *utilis*, 26 unigenes sequences encoding putative ABC transporters were identified (S5 Table). After removing short sequences, 22 unigenes were subjected to phylogenetic analysis with *B*. *dorsalis* and the results showed that these unigenes were divided into six subfamilies: five belong to the ABCB subfamily (22.73%), four belong to the ABCC (18.19%), one belong to the ABCD (4.55%), three belong to the ABCF (13.63%), nine belong to the ABCG (40.9%). No ABC transporters belong to the ABCA and ABCH subfamily (Fig 10). Genome-wide analysis of ABC transporters in the *B*. *dorsalis*, *D*. *melanogaster*, *A*. *gambiae*, *T*. *castaneum*, *A*. *mellifera*, *B*. *mori*, *Daphnia pulex*, *Tetranychus urticae*, *Bemisia tabaci* and *Laodelphax striatellus* led to the identification of 47, 56, 52, 73, 41, 55, 65, 103, 55 and 40 ABC transporters, respectively (Table 4) [80, 84, 85]. Furthermore, in the *B*. *dorsalis* fat body transcriptome, 29 ABC transporters were identified and assigned to 7 subfamilies, including ABCA (4), ABCB (4), ABCC (1), ABCD (2), ABCE (1), ABCF (3), ABCG (4) [13]. Compared with number of ABC transporters in other insect species, there were fewer ABCs in *P*. *utilis*, especially in the ABCA, ABCD and ABCH subfamilies (Table 4). The missing ABC genes in the current transcriptome database may await further discovery.

**Table 4:** Gene numbers in ABC subfamilies of twelve arthropod species.

ABC subfamily	Pu	Bd	Dm	Bo	Ag	Tc	Am	Bm	Dp	Tu	Bt	Ls
A	0	7	10	3	9	10	3	9	4	9	8	2
B	5	7	8	4	5	6	5	5	7	4	3	6
C	4	9	14	2	13	35	9	9	7	39	6	5
D	1	2	2	1	2	2	2	2	2	2	2	2
E	0	1	1	1	1	1	1	1	1	1	1	1
F	3	3	3	3	3	3	3	3	3	3	3	2
G	9	15	15	4	16	13	15	13	24	23	23	14
H	0	3	3	0	3	3	3	3	15	22	9	8
Total	22	47	56	18	52	73	41	55	65	103	55	40

Pu, *P*. *utilis*; Bd, *B*. *dorsalis*; Dm, *D*. *melanogaster*; Ag, *A*. *gambiae*; Tc, *T*. *castaneum*; Am, *A*. *mellifera*; Bm, *B*. *mori*; Dp, *D*. *pulex*; Tu, *T*. *urticae*; Bt, *B*. *tabaci*; *L*. *Striatellus*. Numbers of other nine species were derived from Dermauw and Van Leeuwen (2014), Xiao *et al* (2018).

ABC transporters have been reported to be involved in the translocation of xenobiotics and phytochemicals. For example, in larvae of *Anopheles stephensi*, the ABC transporters were involved in the detoxification of permethrin [86]. In *B*. *dorsalis*, *BdABCF1*, *BdABCF3*, *BdABCG1*, *BdABCG11*, *BdABCH1*, and *BdABCH2* genes were upregulated after treatment with three different pesticides; however the expression level of *BdABCB7* was significantly higher after treatment with malathion than that after treatment with beta-cypermethrin and avermectin, and the toxicity of malathion to *B*. *dorsalis* increased after *BdABCB7* was silenced [85]. In *B*. *tabaci*, the ABC transporters responsed to imidacloprid, a neonicotinoid insecticide, and most of the significantly up-regulated ABC transporters of *B*. *tabaci* (80%) involved in the detoxification of imidacloprid belong to the ABCG family [87]. In addition, the ABC transporters were regulated by heavy metals and implicated in the biochemical detoxification of zinc and copper in *D*. *melanogaster* [62]. Likewise, in the cotton bollworm, *Helicoverpa armigera*, the ABC transporters were associated with the degradation of plant secondary metabolites [88]. Previous studies have shown that most of the ABC transporter genes of other insects involved in detoxification belonged to the three families: A, B and C [13, 86]. Here we identified 22 ABC transporters in the fat body of *P*. *utilis* for the first time and more than half of them also belong to A, B and C families. This was the first analysis of the ABC transporters. It remains to be further studied whether these ABC transporters play roles in the adaptation of *P*. *utilis* to the toxic host *E*. *adenophorum*.

### Identification of the putative immunity-related genes

The insect fat body acts as an immune barrier between the internal and external environment, which plays a major role in preventing infection by microbial, protozoan pathogens and other foreign objects [12, 13, 89]. In recent studies, a number of genes associated with immune response were identified from the fat body transcriptome of *B*. *dorsalis*, *G*. *morsitans morsitans* and *A*. *aegypti*, including lysozyme, serpin, antimicrobial peptide and other genes [13–15, 90]. In this study, 17 putative serpin, five peptidoglycan recognition proteins (PGRP) and four lysozyme unigenes were identified, which played important roles in insect immune defense.

**Fig 11:**
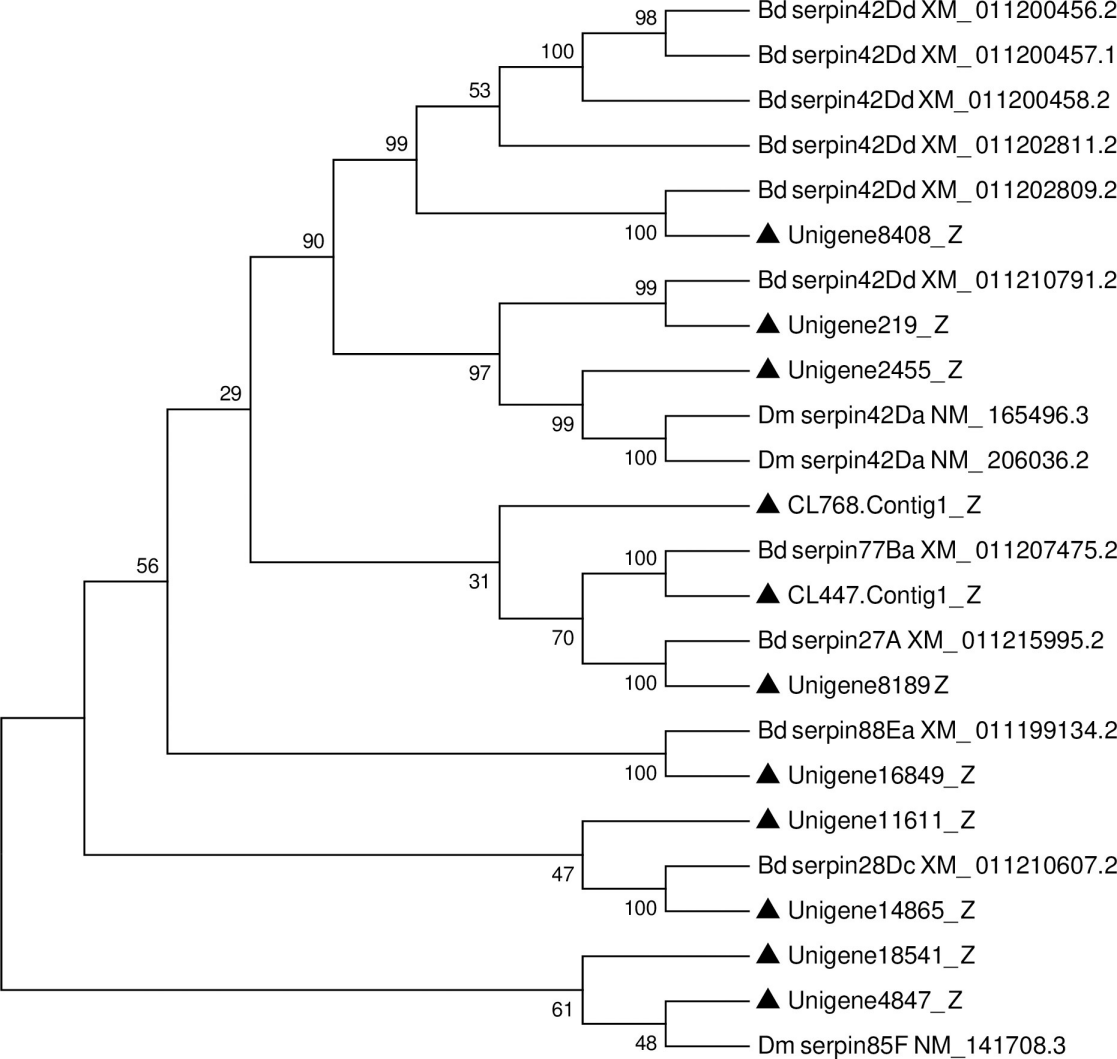
Neighbor-joining phylogenetic analysis of serpin genes from the *P*. *utilis* fat body (▲) and other insects. Numbers at each branch node represent bootstrap values.

Serpins are the largest family of serine proteinase inhibitors with members spread over eukaryotes and prokaryotes. At present, many serpin genes have been identified in a number of insect genomes and transcriptomes (S6 Table) [91–96]. The fat body is a main site for the serpin synthesis, indicating that this organ participates in the immune response of insects. For example, in *Manduca sexta*, serpin-9 and -13 genes were mainly produced in fat body [97]. In this study, a total of 17 putative serpin unigenes were identified (S7 Table). This number was less than in the fat body transcriptome of *B*. *dorsalis* (25) [13]. After removing short sequences, 11 unigenes were used for phylogenetic analysis. The results showed that these unigenes were divided into different clades (Fig 11). Previous studies have illustrated that serpins play vital roles in regulation of innate immune responses via inhibition of serine proteinase cascade pathways. Serpin-5 and serpin-9 two genes in *H*. *armigera* controlled melanization by directly inhibiting their target proteases clip-domain serine protease 4 and clip-domain serine protease 6, respectively [98].

**Fig 12:**
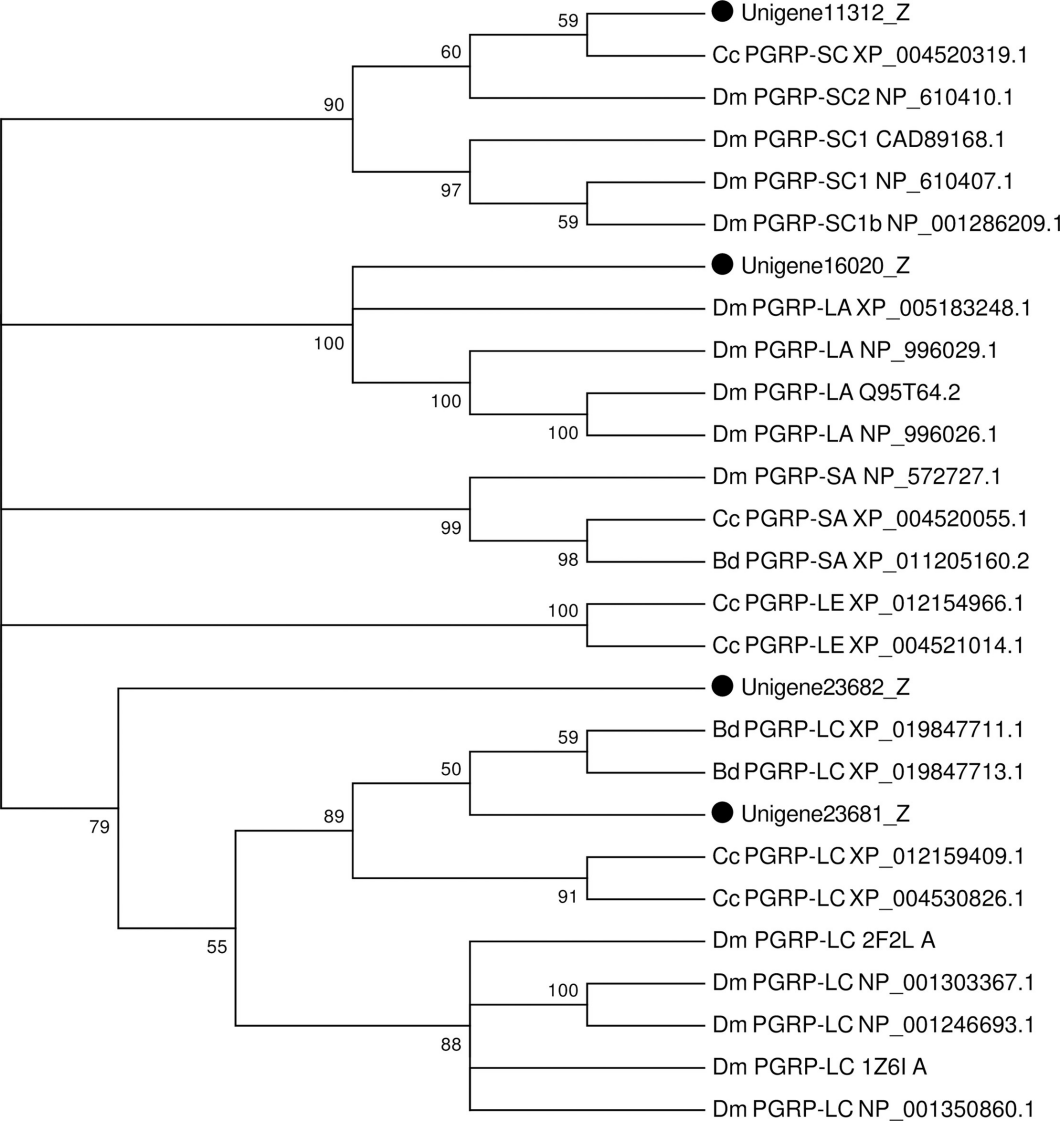
Neighbor-joining phylogenetic analysis of PGRP genes from the *P*. *utilis* fat body (●) and other insects. Numbers at each branch node represent bootstrap values.

Peptidoglycan recognition proteins (PGRPs) form a large family of proteins that recognize peptidoglycan (PGN) in both gram-negative and gram-positive bacteria cell walls and induce the activation of immune responses [99, 100]. The PGRPs are widely distributed and highly conserved from invertebrates to mammals [100]. According to the length of corresponding mRNA, insects PGRPs are divided into two categories: long-type PGRP (PGRP-L) that encodes secreted proteins and short-type PGRP (PGRP-S) that encodes transmembrane or intracellular products, which play distinct roles in innate immune response [101]. In *Drosophila*, PGRP-SA and PGRP-SD can recognize lysine-type peptidoglycan and activate the Toll pathway, while PGRP-LE is essential for activation of the prophenoloxidase (proPO) cascade [102]; DAP-type peptidoglycan is recognized by PGRP-LC and PGRP-LC can interact with PGRP-LE to trigger Imd pathway signaling activation [101]. In *Anopheles*, PGRPLC activates the immune-deficiency (Imd) pathway to resist microbiota load and *Plasmodium* infection [103]. At present, many PGRP genes have been identified from *Drosophila*, *T*. *castaneum*, *A*. *gambiae*, *B*. *dorsalis* and other insects (S6 Table) [13, 104–106]. In this study, five PGRP genes were identified from the fat body transcriptome of *P*. *utilis*, of which one belongs to the short (S) subfamily (PGRPS1, S2, and S3), while four belong to the long (L) subfamily (PGRPLA, LB, LC, and LD) based on the phylogenetic analysis with genes from other insects (S8 Table and Fig 12). The number of PGRP genes in *P*. *utilis* was similar to the number identified in the fat body of *B*. *dorsalis* [13].

The innate insect immune system includes humoral and cellular immunity that can be activated by invasion of pathogens [107]. An array of potent antimicrobial peptides and proteins (AMPs), lysozymes and lectins make up the humoral response. Lysozyme is well-known as an immune effector that is found in both vertebrates and invertebrates, which play protective roles in defense against infections [107, 108]. To date, with genomic and transcriptomic analysis many lysozyme genes have been identified from several insects such as *Galleria mellonella*, *T*. *castaneum*, *Reticulitermes speratus*, *M*. *sexta*, *Acyrthosiphon pisum* and *Harmonia axyridis* (S6 Table) [108–113]. Previous reports have shown that insect lysozyme is synthesized in hemocytes and much more so in the fat body, and the lysozyme is released into the hemolymph [107, 114]. Generally insect lysozyme genes are significantly expressed but upregulated in response to microbial challenge in specific tissues, mainly the fat body and hemocytes [107, 115]. Six hours after injection with *E*. *coli*, *SgLys* transcripts were upregulated 2-fold in the fat body of *Schistocerca gregaria*, indicating that *SgLys* plays a role in *S*. *gregaria* innate immunity [107]. Low levels of transcripts were observed in the fat body of non-infected larvae of *M*. *sexta*, while after treatment with peptidoglycan the levels of transcripts increased rapidly and remained elevated for several days [107, 116].

**Fig 13:**
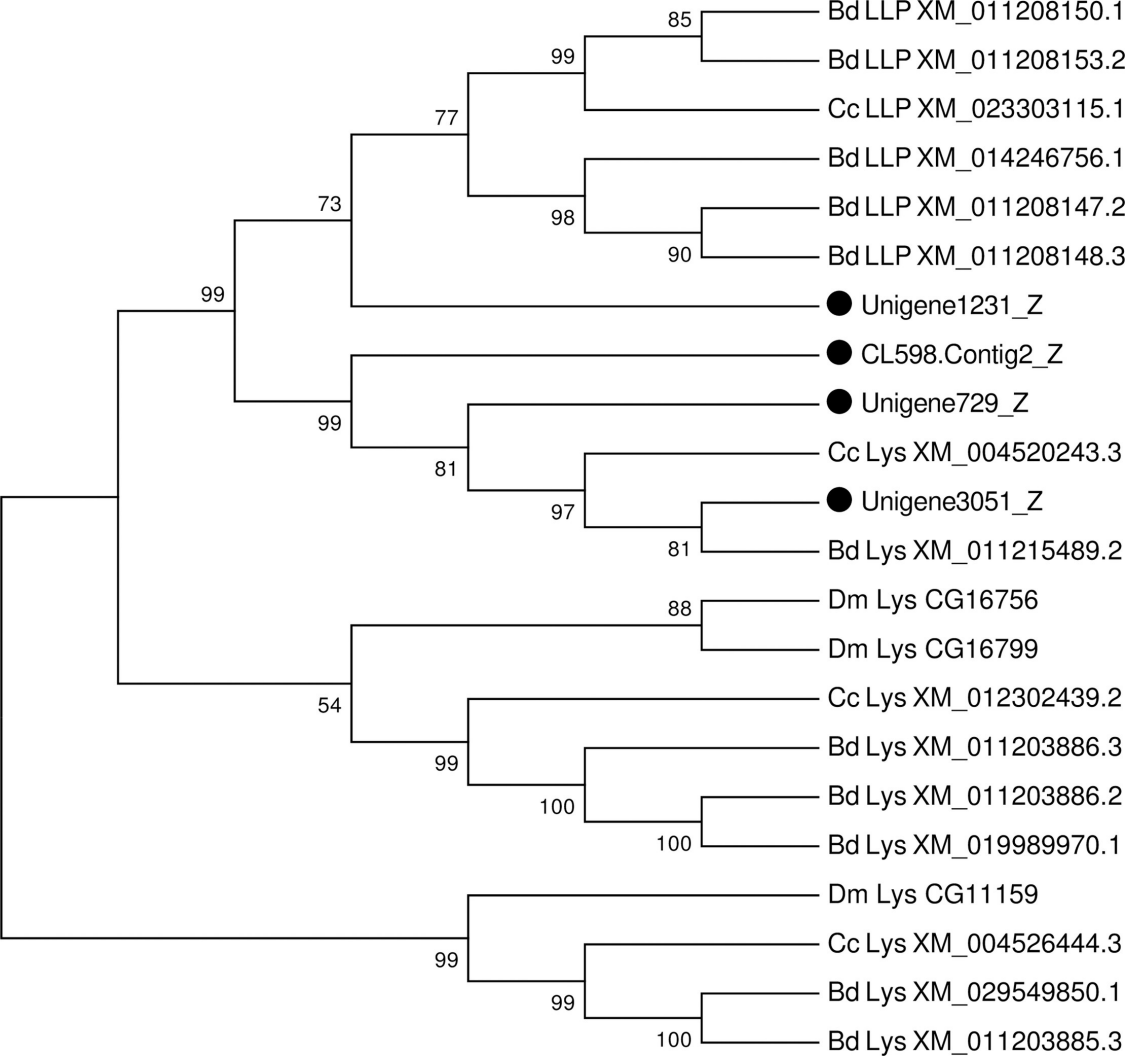
Neighbor-joining phylogenetic analysis of lysozymes and lysozyme-like proteins from the *P*. *utilis* fat body (●) and other insects. Numbers at each branch node represent bootstrap values.

We identified four gene sequences encoding putative lysozymes in the fat body transcriptome of *P*. *utilis*. Among them, three belong to lysozyme genes and one encodes a lysozyme-like proteins (LLPs) gene according to the phylogenetic analysis with other insects (S9 Table and Fig 13), which is similar to or less than the number found in other insects (S6 Table) [91, 106, 108, 110, 117]. LLPs exhibit antimicrobial activity against both gram-positive and gram-negative bacteria, which probably inhibit bacterial membrane, but do not dissolve the cell wall [118]. Lysozyme not only participates in immunity, but also has a digestive function. In *D*. *melanogaster* two lysozyme genes were recruited for the digestion of symbiotic bacteria in the stomach [119]. Therefore, whether lysozymes in *P*. *utilis* are involved in immunization needs further study. This is the first analysis of the lysozymes in the fat body transcriptome of *P*. *utilis*.

### Energy metabolism functions of fat body

The fat body plays major roles in insect metabolism and nutrient storage, which is similar to the liver and fat tissue of vertebrates. The fat body is distributed all over the insect body, but mainly in the abdomen and under the epidermis [10]. Insects store energy in the form of glycogen, triglycerides and amino acids, which form storage proteins in fat body. It is a major storage depot for nutrients stores, and releases energy in response to the energy demands of the insect [10, 13]. In this study, we found many unigenes that are highly similar to those related to energy metabolism in other insects, including 18 lipases, five fatty acid synthase and six elongases of very long chain fatty acid (ELOVL).

**Fig 14:**
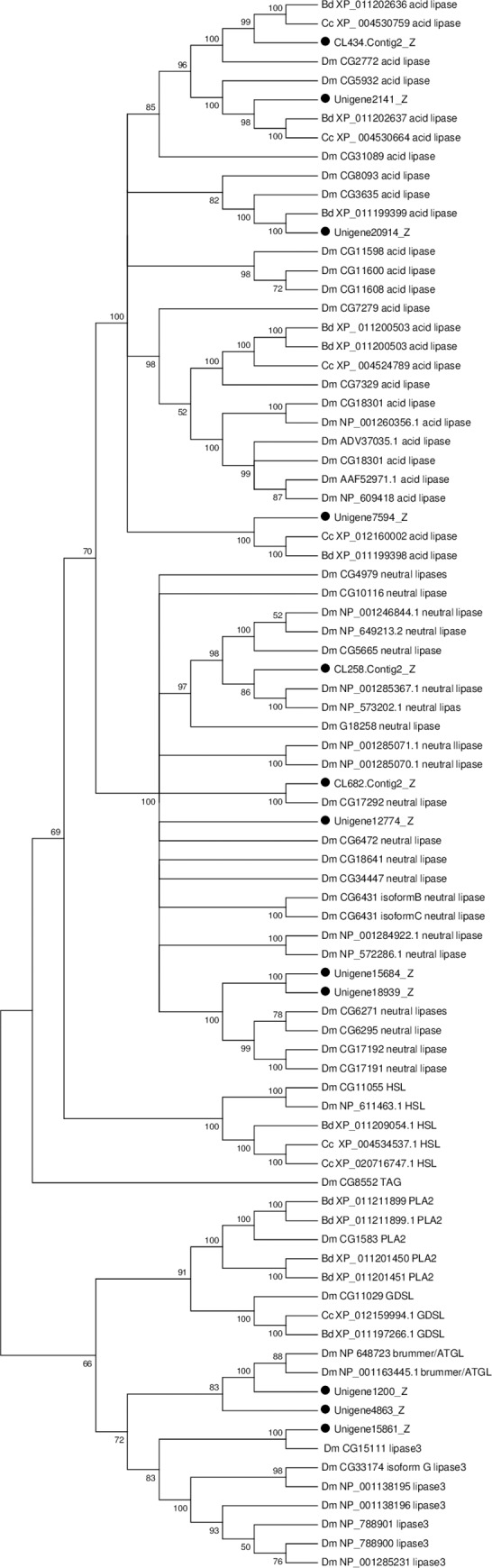
Neighbor-joining phylogenetic analysis of lipases genes from the *P*. *utilis* fat body (●) and other insects. Numbers at each branch node represent bootstrap values.

Lipids are the main component of the fat body and the major source of metabolic energy [13]. Lipases are a special type of serine hydrolase, which are widely found in vertebrates and invertebrates and play a crucial role in fat metabolism [14]. According to sequence relationships within the α/β hydrolase fold superfamily of proteins, lipases are divided into six families: neutral (Pfam: PF00151), acid (Pfam: PF04083), lipase 2 (Pfam: PF01674), lipase 3 (Pfam: PF01764), GDSL (Pfam: PF00657) and hormone sensitive lipases (HSL) [120, 121]. The genes encoding lipases were identified in the genomes of many insects (S6 Table) [121, 122]. According to our research on the *P*. *utilis* fat body transcriptome, a total of 18 unigenes putatively encoding lipase were identified (S10 Table), which is less than the number found in other insects [121, 122]. After manually removing short sequences, 12 unigenes were used for the phylogenetic analysis and the result indicated that they were divided into four families, five belonged to the neutral lipase, four belonged to the acid lipase, two belonged to the Brummer and one belonged to the lipase 3. No unigene belonged to lipase 2 and hormone sensitive lipases (Fig 14). The features and functions of lipase vary with families. In insects, neutral lipases have been characterized to play important roles in regulating lipolysis in the fat body. For example, a triacylglycerol lipase with phospholipase A1 activity in the fat body of *M*. *sexta* plays a crucial role in regulating lipolysis [10, 123]. Brummer lipase, or insect adipose triglyceride lipase (ATGL) has been identified in some insects such as *D*. *melanogaster*, *B*. *mori* and *G*. *morsitans* [121, 123–125]. During starvation, Brummer is crucial for lipolysis in the fat body of *Drosophila* [124]. Furthermore, a mutation of Brummer in *Drosophila* reduced fat body lipid accumulation, nevertheless overexpression of Brummer results in lean flies [123, 124]. The acid lipase family includes gastric lipases, pregastric or lingual lipase, pregastric esterase and lysosomal acid lipases (LAL) [126], which function in the intracellular hydrolysis of cholesteryl esters and triglycerides that are internalized through receptor mediated endocytosis of lipoprotein particles.

**Fig 15:**
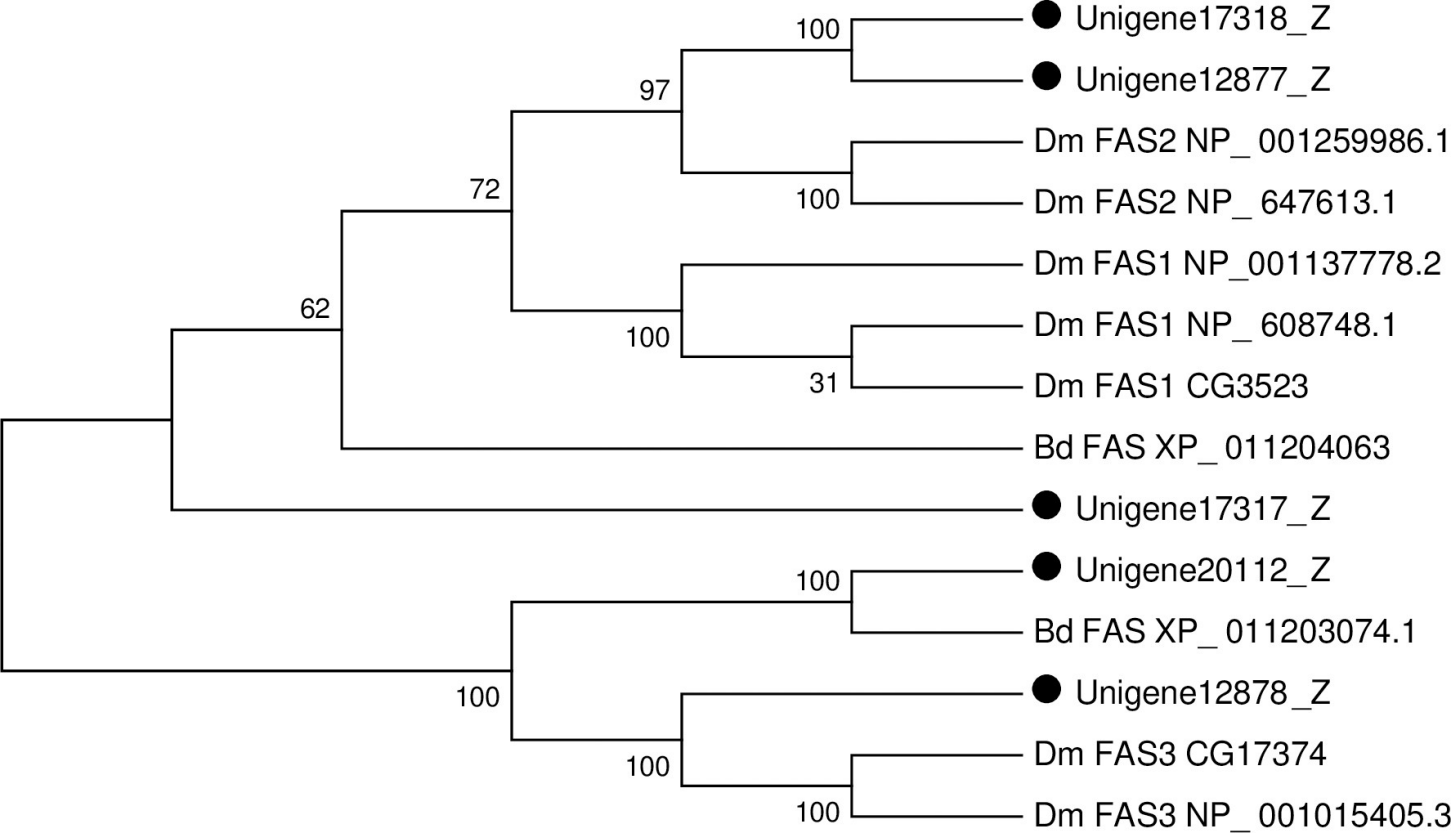
Gene identification of *P*.*utilis* FASs. Numbers at each branch node represent bootstrap values.

Lipids accumulated as fat reserves in the fat body provide energy for insect growth and development. Fatty acid synthase (FAS) plays an important role in lipid synthesis, which is a key enzyme for biosynthesis of endogenous fatty acid in both vertebrates and invertebrates [127]. In *D*. *melanogaster* genome, three distinct type I FAS genes (*CG3523*, *CG3524* and *CG17374*) were identified [122]. RNA *in situ* hybridization of these three genesin *D*. *melanogaster* adult males showed that *CG3523* was expressed only in the adult fat body and the other two were expressed in oenocytes [122, 128]. Three FAS I genes (*RPRC00269*, *RPRC002909* and *RPRC000123*) were identified from the *R*. *prolixus* genome [122]. In this study, a total of five unigenes putatively encoding FAS were identified (S11 Table). Phylogenetic analysis with genes from other insects was carried out, and two unigenes (Unigene17317_Z, Unigene17318_Z) showed homology with FAS1 from *B*. *dorsalis*, *D*. *melanogaster* and *A*. *aegypti*. Unigene12878_Z and Unigene20112_Z were exclusively detected in *Drosophila* oenocytes (*CG17374)* and *B*. *dorsalis* FAS gene, respectively (Fig 15). It has been reported that biochemical deficiencies in FAS1 inhibit *de novo* lipid biosynthesis in *A*. *aegypti* blood fed mosquitoes, as well as impact eggshell formation and blood digestion [10]. Moreover, when FAS1 expression was knocked down at mRNA levels inhibition of fatty acid biosynthesis in *A*. *aegypti* blood fed mosquitoes by RNAi [10]. In *Colaphellus bowringi*, FAS plays a central role in lipid metabolism during diapause, and knockdown of FAS2 reduced lipid accumulation as well as affected stress tolerance genes expression and water content of this species [127].

**Fig 16:**
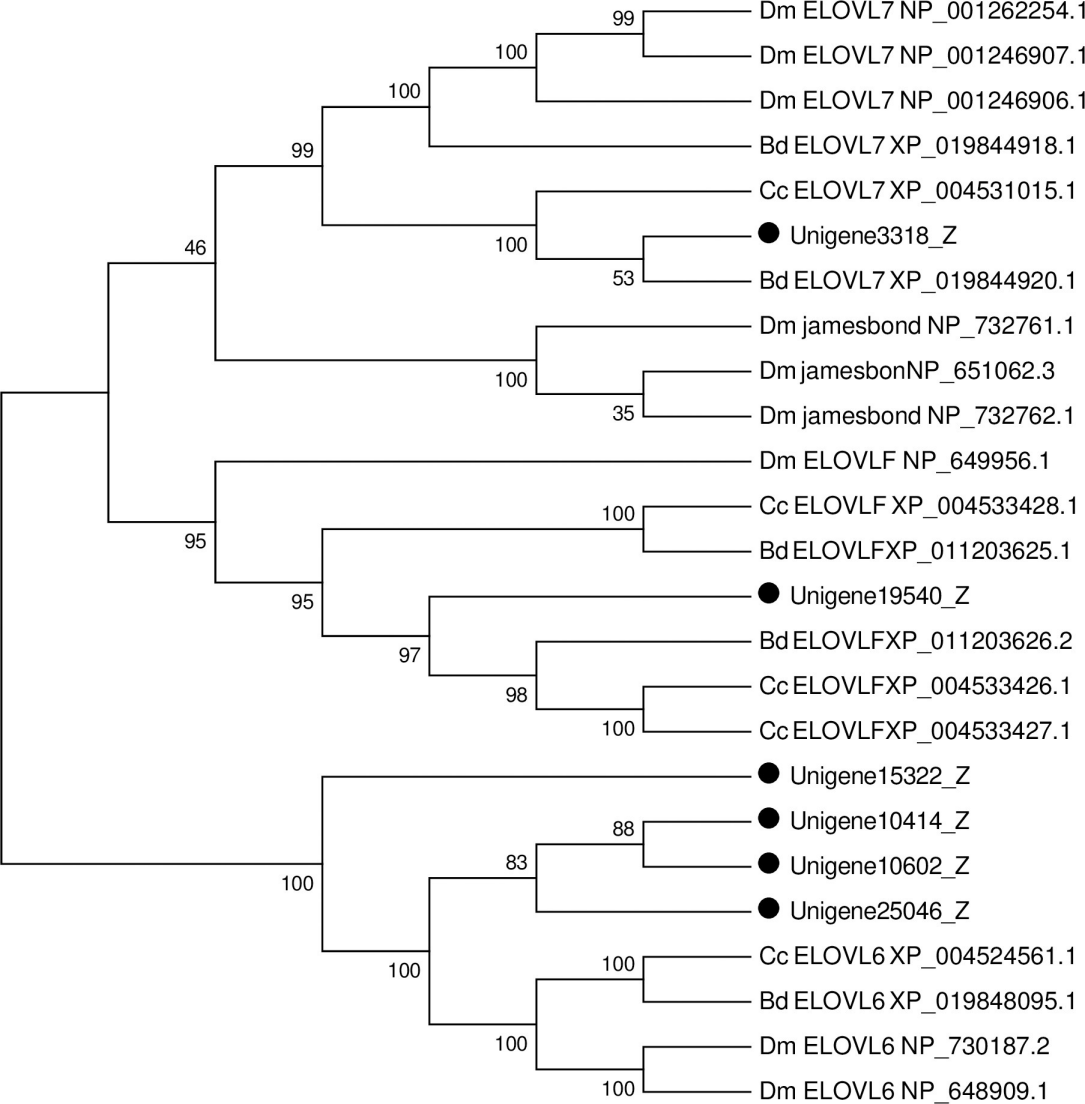
Neighbor-joining phylogenetic analysis of ELOVL genes from the *P*. *utilis* fat body(●) and other insects. Numbers at each branch node represent bootstrap values.

Both FAS products and fatty acids taken up from the diet are further elongated into very long chain fatty acids (VLCFA) [122]. Elongases of very long chain fatty acid (ELOVL) are enzymes that are essential for the biosynthesis of VLCFAs and elongation of long-chain fatty acids [129]. ELOVLs, which are conserved from microorganisms to mammals, play key roles in various biological processes [130]. In insects, a large number of ELOVL genes have been identified from their genomes although most of their functions remain mostly unknown, including *D*. *melanogaster*, *B*. *mori* and *R*. *prolixus* [122, 129, 131]. In the present study, we obtained a total of six unigenes encoding ELOVL proteins from the *P*. *utilis* fat body transcriptome (S12 Table). All of these unigenes were used for phylogenetic analysis with other insects. Phylogenetic analysis revealed that they divided into three clades: ELOVL 3/6, ELOVL 1/7 and “ELOVL X clades” (Fig 16). No genes belong to ELOVL 2/5 and ELOVL 4 clade. The “ELOVL 1/7 clade” includes the *P*. *utilis* genes Unigene3318, this clade possibly plays an important role in elongating saturated/monounsaturated fatty acids. Within these clades, functional characterization is available for the mosquito *Aedes albopictus* (*ACS37245*), which play a critical role to control the dehydration resistance of diapause eggs by regulating hydrocarbon formation from VLCFA precursors [132]. Four ELOVL unigenes of *P*. *utilis* (Unigene15322, Unigene25406, Unigene10414 and Unigene1062) belong to the ELOVL 3/6, this clade includes an ELOVL gene of the fruit fly gene *baldspot* (*CG3971*) (Fig 16), which is involved in the sex pheromone production, viability, and sperm development [131]. The remaining unigene (Unigene19540) in the insect-specific ELOVL clusters “ELOVL X clades,” which related to both the ELOVL 1/7 and ELOVL 4 clades (Fig 16). This clade includes two ELOVL genes of the fruit fly: elongase F (*CG16905*) and james bond (*CG6921*), which are also involved in sex pheromone production, viability, and sperm development [131].

## Conclusions

Here we report the first comprehensive analysis of the fat body transcriptome of *P*. *utilis* using Illumina sequencing technology. We assembled the raw reads into 30,559 unigenes with an average length of 539 bp. Among these unigenes, 21,439, 13,657, 12,188, 12,405, 12,925, 13,149 and 9,354 unigenes matched to Nr, Nt, SwissProt, KEGG, KOG, InterPro and GO databases, respectively. A number of unigenes that are associated with detoxification (P450s, GSTs, CarEs and ABC transporters), immunity (serpin, PGRPs and lysozymes) and energy metabolism (lipase, FAS and ELOVL) were identified from the annotated unigenes. Specific information of these unigenes identified in this study is shown in S2–S12 Tables. In addition, the fat body-specific transcriptome analysis generated a great deal of unigenes newly identified in *P*. *utilis*, which provided a major genomic resource for investigating the fat body of *P*. *utilis* and laid the foundation for future functional genomics studies.

## Supporting information

S1 Table Distribution of simple sequence repeat (SSR) types found in the *Procecidochares utilis* transcriptome unigenes. (XLSX)

S2 Table Unigene sequences for cytochrome oxidase P450s identified in the *Procecidochares utilis* transcriptome. (XLSX)

S3 Table Unigene sequences for the glutathione s-transferases identified in the *Procecidochares utilis* transcriptome. (XLSX)

S4 Table Unigene sequences for carboxylesterases identified in the *Procecidochares utilis* transcriptome. (XLSX)

S5 Table Unigene sequences for ATP-binding cassette (ABC) transporters identified in the *Procecidochares utilis* transcriptome. (XLSX)

S6 Table Number of serpins, PGRPs, lysozymes and lipases in other insects. (XLSX)

S7 Table Unigene sequences for serpins identified in the *Procecidochares utilis* transcriptome. (XLSX)

S8 Table Unigene sequences for peptidoglycan recognition proteins identified in the *Procecidochares utilis* transcriptome. (XLSX)

S9 Table Unigene sequences for lysozymes identified in the *Procecidochares utilis* transcriptome. (XLSX)

S10 Table Unigene sequences for lipases identified in the *Procecidochares utilis* transcriptome. (XLSX)

S11 Table Unigene sequences for fatty acid synthases identified in the *Procecidochares utilis* transcriptome. (XLSX)

S12 Table Unigene sequences for elongases of very long chain fatty acids identified in the *Procecidochares utilis* transcriptome. (XLSX)

S1 Text (TXT)
